# Clique-Finding for Heterogeneity and Multidimensionality in Biomarker Epidemiology Research: The CHAMBER Algorithm

**DOI:** 10.1371/journal.pone.0004862

**Published:** 2009-03-16

**Authors:** Richard A. Mushlin, Stephen Gallagher, Aaron Kershenbaum, Timothy R. Rebbeck

**Affiliations:** 1 PsychoGenics Inc., Tarrytown, New York, United States of America; 2 Center for Clinical Epidemiology and Biostatistics, University of Pennsylvania School of Medicine and Abramson Cancer Center, Philadelphia, Pennsylvania, United States of America; 3 IBM T.J. Watson Research Center, Yorktown Heights, New York, United States of America; Karolinska Institutet, Sweden

## Abstract

**Background:**

Commonly-occurring disease etiology may involve complex combinations of genes and exposures resulting in etiologic heterogeneity. We present a computational algorithm that employs clique-finding for heterogeneity and multidimensionality in biomedical and epidemiological research (the “CHAMBER” algorithm).

**Methodology/Principal Findings:**

This algorithm uses graph-building to (1) identify genetic variants that influence disease risk and (2) predict individuals at risk for disease based on inherited genotype. We use a set-covering algorithm to identify optimal cliques and a Boolean function that identifies etiologically heterogeneous groups of individuals. We evaluated this approach using simulated case-control genotype-disease associations involving two- and four-gene patterns. The CHAMBER algorithm correctly identified these simulated etiologies. We also used two population-based case-control studies of breast and endometrial cancer in African American and Caucasian women considering data on genotypes involved in steroid hormone metabolism. We identified novel patterns in both cancer sites that involved genes that sulfate or glucuronidate estrogens or catecholestrogens. These associations were consistent with the hypothesized biological functions of these genes. We also identified cliques representing the joint effect of multiple candidate genes in all groups, suggesting the existence of biologically plausible combinations of hormone metabolism genes in both breast and endometrial cancer in both races.

**Conclusions:**

The CHAMBER algorithm may have utility in exploring the multifactorial etiology and etiologic heterogeneity in complex disease.

## Introduction

Under the common gene, common disease hypothesis[1], commonly occurring diseases may result from the effects of multiple genetic and environmental exposures, often involving complex biochemical pathways. Currently, genome-wide association studies (GWAS) are identifying common variants that confer low risk of complex disease. The success of these efforts means that new approaches will be required to follow up on these gene discovery efforts to better evaluate higher-order relationships among genotypes and other risk factors. A number of methods have already been proposed that could begin to achieve this goal, including recursive partitioning[2], [3] and related methods such as those implemented in the FlexTree program[4]; random forests[5]; combinatorial partitioning[6]; multifactor-dimensionality reduction[7]; permutation-based procedures[8]; multivariate adaptive regression spines[9]; boosting [10]; support vector machines[11]; neural networks[12]; “Detection of Informative Combined Effects” (DICE)[13], [14]; Bayesian pathway modeling approaches[15], [16]; the Relief, ReliefF, and ‘tuned’ ReliefF (turf) algorithms[17], [18], [19]; partial linear tree regression[20]; algorithms adapted from gene expression analysis, such as GenesWork [21]; logic regression[22]; penalized logstic regression approaches [23]; and other greedy algorithms for combinatoric searches involving multiple genotypes [24]. Traditional machine learning techniques [25] can also applied to problems where the number of factors being considered is relatively small in comparison to the number of data samples available and their running times tend to be non-linear in the number of factors. Similarly, agglomerative clustering approaches have also been proposed that would place a single allele into one cluster and then combine clusters based on some objective function. Advantages of these approaches include: the ability to detect effects of higher-order genotype combinations when there is no main effect of individual genes; avoiding P-value-based hypothesis testing (see Appendix S1, Step 3) and the associated power/sample size limitations for exploratory purposes; limited assumptions of interactive effects (i.e. multiplicative) of predictor variables; and no assumption of linear effect of predictor variables. However, most of these approaches make assumptions about the underlying biological model of disease, require assumptions about the identification of “purity” in the groupings identified, or may miss combinations that are not consistent with the hierarchical nature of nodes due to the use of “greedy” algorithms. Therefore, there is room for additional methodology to address questions of complex disease etiology. In addition, existing methods tend not to consider the likely heterogeneity in etiology of disease, defined as the existence of two or more explanations for the occurrence or pattern of disease in a population. Previous methods have been proposed to assess etiologic heterogeneity in complex disease [26], [27], [28], [29].

Our goal is to develop computational methods to explore higher-order relationships between groups of predictor variables that discriminate between cases and controls. This approach identifies combinations of genotypes and epidemiologic risk factors that may identify risk groups, and identifies etiologically heterogeneous subgroups of individuals in the population whose risk is determined by different combinations of risk factors. To accomplish this, we undertake an exhaustive exploration of all possible combinations of risk factors, identify “bi-cliques” that contain all individuals that have the particular risk factor combinations, compute a Figure of merit (FOM) that quantifies risk, build hierarchies (lattices) of bi-cliques that define risk sets. Finally, we use this information to define groups of individuals whose risk is defined by specific bi-cliques, thus identifying etiologic heterogeneity in the population defined by different combinations of the same set of variables, different variable sets, or both. We apply this approach to both simulated data and empirical data on candidate estrogen metabolism genotypes in two case control studies of breast and endometrial cancer[30], [31]. We present an analytical procedure that thoroughly explores the complete space of combinations among all factors considered and is not dependent on the order in which the variables enter the algorithm. Unlike most hypothesis-testing strategies, a key feature of this algorithm is that it allows the user to explore complex etiological relationships in data rather than serve as a tool to find the “best” result. We consider combinations of alleles or genotypes that result in very large numbers of groupings. By considering bi-cliques, we take into account many interrelationships among the alleles and are able to make use of a procedure that only needs to look at each combination a very small number of times, thereby keeping the computational effort manageable despite the large number of combinations.

## Results

### Simulated Data

**Figure 1:**
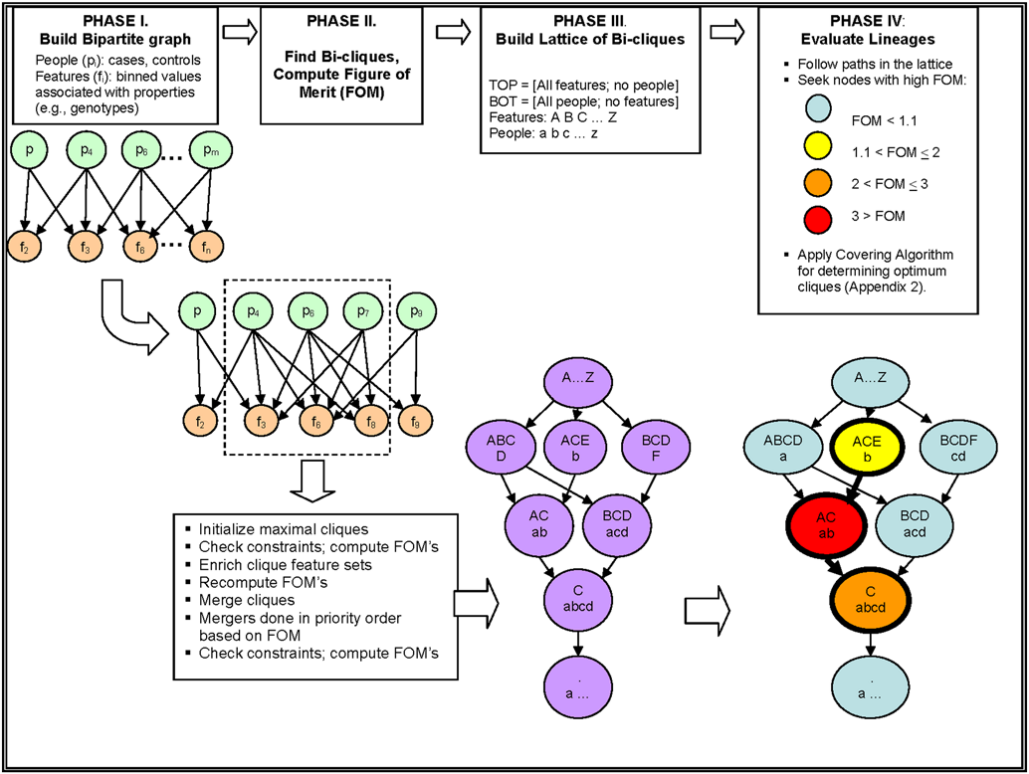
Overview of the Bi-clique-Finding Algorithm. Step 1 involves the construction of bipartite graph to identify all relationships between nodes (Figure 1, Phase I). In Step 2, the algorithm undertakes maximal bi-clique formation by exhaustively searching the entire space of all genotype combinations to identify an initial set of maximal bi-cliques (Figure 1, Phase II). In the third step, a Figure of merit (FOM) is generated to prioritize “interesting” bi-cliques (Figure 1, Phase II). The FOM can be any measure inherent to the data. Here, we consider values of features (e.g., genotypes) in a 2×2 contingency Table with affected cases and unaffected controls contingent on exposure (e.g., genotype). In the fourth step, a “lattice” is built by connecting each pair of bi-cliques to their least upper bound and their greatest lower bound using principles of set union and intersection. (Figure 1, Phase III). In the fifth step, the bi-cliques of greatest interest are identified using a parsimony principle by which “optimal” bi-cliques should contain the most parsimonious set of features, and the addition of more features does not substantially improve the FOM. To achieve this, we employ the set covering approach[33] (Appendix S1).

**Figure 2:**
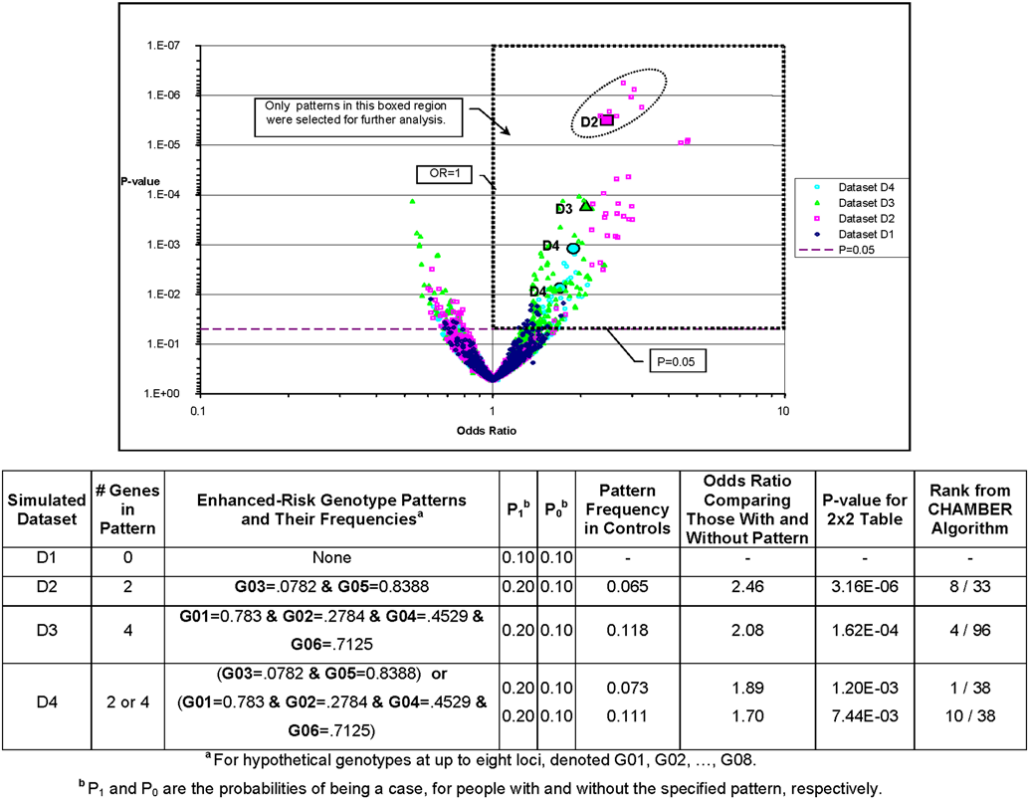
Distribution of P-values and Odds Ratios for Four Simulated Datasets. Designated patterns in D2–D4 are shown as large filled glyphs. Dataset D1 was modeled to have no factors that confer risk of being a case vs. a control. Datasets D2 and D3 contain a 2-gene and a 4-gene risk pattern respectively. Dataset D4 simulated the situation of etiologic heterogeneity in which disease risk was conferred by different patterns in different subsamples. The list of all discovered patterns was filtered to include only those with support>5% of cases, odds ratio>1, and P-value<0.05. P-value was used as the FOM. Note that adding even a single high risk genotype (D2, D3) results in many patterns above the noise level (D1).

Synthetic data were generated for analysis by the CHAMBER (Figure 1). Simulated data were generated for eight genes to reflect the empirical dataset described below [30], [31], [32] (Figure 2). Dataset D1 had no factors that confer risk of being a case vs. a control. Datasets D2 and D3 contain a 2-gene and a 4-gene risk pattern, respectively. Dataset D4 simulated etiologic heterogeneity in which disease risk was conferred by different patterns in different subsamples. We refer to bi-cliques, which are a set of alleles (features) together with a set of people (cases and controls) sharing these alleles. We refer to the set of alleles as a pattern and to the set of people as a support for the pattern. In some cases, where only the set of alleles is focused on, we use the terms pattern and bi-clique interchangeably.

**Figure 3:**
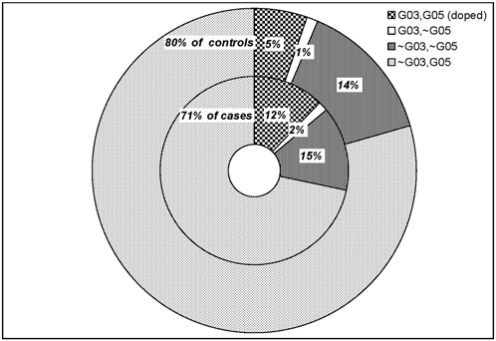
Dataset D2 partitioned by the 2 genes in the designated pattern for cases (inner band) and controls (outer band). The solid white sector represents the single feature G03 *without* G05. The checkered sector represents G03 *with* G05. So the checkered and white sector together represent all the people with G03. One can see that generalizing the description of the risky pattern from G03 *and* G05 to simply G03 identifies *all* the people with the high risk 2-gene pattern, while picking up only a small fraction of low risk false positives. Frequencies are rounded to 1%, and the “∼” symbol represents logical “not”.

Figure 2 shows the relationship between the patterns simulated to have increased risk and all other patterns that were not simulated in the data to have increased risk. In dataset D1 (no genetic risk), very few patterns had OR>1.5 or P<0.05. In datasets D2–D4, patterns simulated to have risk-increasing effects are among the best in both odds ratio and P-value. The algorithm identified those patterns that were simulated to have increased risk (solid symbols; Figure 2), and high-scoring patterns that were not simulated to have risk-increasing effects (i.e., higher-scoring patterns depicted with hollow symbols; Figure 2). This phenomenon is expected to occur because the features that are contained in the simulated high-risk bi-cliques are also contained in other bi-cliques, and thus may cause those bi-cliques to have high scores as well. This is not a limitation of the algorithm but the expected result when considering complex relationships among risk factors. To illustrate this point, Figure 3 depicts the frequencies of the four partitions created by the two features G03 and G05 for cases (inner ring) and controls (outer ring). The areas of the rings represent the relative sizes of the case and control populations. These data indicate that bi-cliques sharing alleles and people in common with the high-ranking bi-cliques will also rank well. Thus, the key to the interpretation of the algorithm is to carefully evaluate the results and determine both the high-ranking bi-cliques as well as their relationships with other (related) bi-cliques.

Since bi-cliques related by shared features can be almost equivalent in their ability to select the population with the greatest risk, we use a set covering technique[33] to identify the most parsimonious bi-cliques that balance high risk prediction with the complexity of the feature set. Set-covering assigns “costs” to potential solutions, and minimizes the total cost of a solution based on a cost model (Appendix S1). One element can “cover” another element if it can logically do so and the cost of doing so is low enough. For example, the genotype pattern AB can cover patterns ABC, ABD, ABCD, etc. But there is an associated cost, namely a possible decrease in FOM that may result from adding irrelevant genotypes. Our model assigns a cost-to-cover based on the FOMs of the two bi-cliques. In the following discussion, we refer to the set of patterns we are trying to cover as the input patterns, and the set of patterns that cover the input patterns using our cost model as the covering patterns.

We applied the set covering procedure[33] (Appendix S1) to simulated datasets (D2–D4). We selected all patterns having OR>1 and P-value<0.05 as the input patterns. A detailed depiction of the analysis results for dataset D2 is presented in Table 1. As summarized in Table 2, Dataset D2 is almost completely covered by the single rare G03 genotype (i.e., 30 covered of 33 input). Dataset D3 yields five covering patterns. Between them, they account for 94/96 of the input patterns, with one of them covering 56 of the original 96 patterns. Note that some of the covering patterns (e.g., the first) could have been covered by other patterns (e.g., the second or the fourth), but CHAMBER determined that the FOMs were too far apart and the cost too high to allow this result. Dataset D4 is covered by two patterns, one for each of the risk components (Table 2). The combined coverage is 33 covered of 38 input. Note that our set covering procedure drastically reduces (by more than an order of magnitude) the number of patterns to be considered for further study.

**Table 1:** Relationships among top-ranking Bi-cliques from Simulated Dataset D2.

Rank	G01 = 0.783	G03 = 0.0782	G05 = 0.8388	G08 = 0.8821	Fisher's Exact Test P-value
1	T	T			5.59×10^−7^
2	S	S	S		7.55×10^−7^
3	T	T		T	1.07×10^−6^
4	S	S	S	S	1.72×10^−6^
5		T		T	2.11×10^−6^
6		T			2.59×10^−6^
7		S	S	S	2.62×10^−6^
8		R	R		3.16×10^−6^

Genes are labeled with their frequencies used for simulating the dataset. The designated high risk pattern, marked R, is ranked 8^th^. Some specializations of R, marked S, are also high risk. Thus, bi-cliques ranked 2, 4, and 7 are specific instances of bi-clique 8, and include 78%, 69%, and 88%, respectively, of the same individuals as bi-clique 8. All confer an approximately two-fold enhanced risk of disease. These patterns all contain the rare allele (7.8%) for G03, plus common alleles of G01, G05, and G08. Thus, the chance of having the designated genotype pattern if the individual has G03 = 0.0782 is 84%, regardless of the genotypes at the other loci. Stated differently, 84% of the individuals in bi-cliques 1, 3, 5, and 6 have the simulated combination of risk-conferring alleles. G03 is the single gene selected by our set covering algorithm to be the most parsimonious description of all the significant risky patterns. Note that patterns containing G03 but *not* G05, marked T, involve very common genes combined with G03. This makes the population at risk from these patterns a large subset of the population described by G03 alone. Similar effects are seen in datasets D3 and D4.

**Table 2:** Summary of Results of Set Covering Algorithm for Simulated Datasets.

Dataset	Designated Risk Pattern	Covering Pattern	Coverage	OR	P
D2	**G03** = 0.0782 **& G05** = 0.8388	**G03** = 0.0782	30/33 (91%)	2.33	2.59E-06
		None	3/33 (9%)		
D3	**G01** = 0.783 **& G02** = 0.2784 **& G04** = 0.4529 **& G06** = 0.7125	**G02** = 0.2784 **& G04** = 0.4529 **& G06** = 0.7125	9/96 (9%)	1.98	1.06E-04
		**G04** = 0.4529	56/96 (58%)	1.39	3.98E-03
		**G02** = 0.1919 **&** G07 = 0.3285	8/96 (8%)	1.78	8.42E-03
		**G02** = 0.2784 **& G06** = 0.7125	14/96 (15%)	1.38	1.37E-02
		G08 = 0.8821	7/96 (7%)	1.56	3.84E-02
		None	2/96 (2%)		
D4	**G03** = 0.0782 **& G05** = 0.8388	**G03** = 0.0782	9/38 (24%)	1.76	2.37E-03
	**G01** = 0.783 **& G02** = 0.2784 **& G04** = 0.4529 **& G06** = 0.7125	**G02** = 0.2784 **& G04** = 0.4529	24/38 (63%)	1.44	1.29E-02
		None	5/38 (13%)		

The set covering algorithm was run on the bi-cliques found in the three simulated datasets. The fraction of input patterns covered by each covering pattern is shown. In dataset D2, 30 of the 33 input patterns could be covered by the single pattern G03 = 0.0782. This is consistent with the data in Table 1, where the common thread of G03 was seen in all eight top patterns. The number of interesting patterns in D2 has been reduced from 30 to 1. Dataset D3 has a more complex risk (four genes), and five patterns were needed to cover 94 of the 96 bi-cliques found in D3. Note that the first cover (3 genes, P≈0.0001) could itself be covered by the second cover (1 gene, P≈0.0040) or the fourth cover (two genes, P≈0.0137). However, the cost model (Appendix S1, Step 5) determined that the difference in P values between these was too large to generalize the three-gene cover pattern to a more parsimonious, but less significant, one- or two-gene cover pattern. Dataset D4, with risk from both the D2 and D3 patterns in the same population, is covered by two simpler patterns. Note that the first D4 cover is the same as the D2 cover. The other D4 cover is a simpler version of the top D3 cover. This slight difference is not unexpected since, for reasons discussed in the text and Appendix S3, the odds ratios and P values are different in the heterogeneous population D4 than in the homogeneous populations D2 and D3.

### Comparison with CART Methods

To compare the CHAMBER method with another approach that has been used to address similar research questions, we have performed a classification and regression tree ( CART) analysis using the simulation data described above and the J48 algorithm with 10-fold cross validation for CART as implemented in the WEKA software[34]. First, using dataset D2 that simulated a 2-gene-risk model, (Table 2), CART split first on gene G03, which was one of the seeded patterns in the data set. However, it was not able to split on the other seeded pattern involving gene G05. Indeed, after splitting on G03, CART did not consistently identify any other seeded pattern in the data that could not be easily trimmed from analysis.

Second, we analyzed dataset D4 (Table 2) that included the pattern found in dataset D2 as well as a second pattern, thus simulating etiologic heterogeneity. CART split first on gene G08, which was not simulated to have any effect in the data set. The second and third splits were on genes G03 and G05, respectively, which were seeded as part of the simulated pattern in the data (Table 2). However, no other clear patterns (splits) were identified. Therefore, in both situations, CART did not identify the simulated data patterns that were correctly identified by CHAMBER. CART appeared to only identify the strongest effects (based on odds ratio estimates, Table 2), and consistently missed weaker effects that were identified by CHAMBER.

### Estrogen Metabolism Genotypes and Breast Cancer

We studied 225 African American (AA) and 613 European American (EA) breast cancer cases, who were compared to 512 AA controls and 820 EA controls. In addition, we studied 44 AA and 462 EA endometrial cancer cases compared with 329 African American and 1,082 White controls who participated in the WISE study. Table 3 presents the results of our empirical data analyses of eight genes involved in estrogen metabolism. In AA breast cancer, the highest scoring bi-clique involved the joint effect of CYP1B1*4 and *UGT1A1* genotypes. The second highest scoring bi-clique also involved these two genes, but also included CYP1A1*2C genotype. The third bi-clique also involved genotypes of *UGT1A1*, but involved the additional effect of *SULT1E1* genotypes. In EA breast cancer, the highest scoring bi-clique involved the joint effect of *CYP1A2* genotypes, *SULT1A1* genotypes, and *UGT1A1* genotypes.

**Table 3:** Results of the CHAMBER Algorithm for the Detection of High-Dimensional Combinations: Estrogen Metabolism Genes in a Population-Based Case-Control Study of Breast and Endometrial Cancer.

Group^a^	Exposed Cases	Exposed Controls	Unexp. Cases	Unexp. Controls	N	OR	P-value	*COMT*	*CYP1A1*	*CYP1A2*	*CYP1B1*	*CYP3A4*	*SULT1A1*	*SULT1E1*	*UGT1A1*
**AA** a **Breast Cancer**	11	4	146	365	526	6.88	0.0005				*AG*				*1*28
	49	71	106	294	520	1.91	0.0022		*AA*		*AA*				*1*1
	41	59	118	312	530	1.84	0.0062							*GG*	*1*1
	57	95	112	292	556	1.56	0.0173								*1*1
	15	17	128	333	493	2.30	0.0206			*CC*			*AG*		
	58	108	115	308	589	1.44	0.0403	*GG*	*AA*					*GG*	
	28	46	131	349	554	1.62	0.0441			*AC*		*GG*		*GG*	
	19	29	108	296	452	1.80	0.0471	*GG*		*AA*			*GG*		
**EA** b **Breast Cancer**	53	39	344	482	918	1.90	0.0025			*AA*			*GG*		*1*28
	51	38	530	740	1359	1.87	0.0030		*AG*						
	78	73	399	589	1139	1.58	0.0060	*GA*			*AG*				
	41	34	463	662	1200	1.72	0.0153			*AC*				*AG*	
	99	105	378	557	1139	1.39	0.0207	*GG*			*AA*				
	115	122	313	435	985	1.31	0.0419			*AA*			*GG*		
**AA Endo-metrial Cancer**	13	56	22	221	312	2.33	0.0237		*AA*		*AA*			*AG*	
**EA Endo-metrial Cancer**	43	58	388	960	1449	1.83	0.0031		*AG*						
	394	918	21	92	1425	1.88	0.0055					*AA*			
	113	210	269	681	1273	1.36	0.0149			*AC*	*AA*				
	35	45	285	621	986	1.69	0.0182	*AA*			*AA*		*GG*		
	43	67	297	687	1094	1.48	0.0371	*GG*							*1*28

a AA = African American.

b EA = European American.

In both races, the algorithm identified combinations of genes involved in phase I catecholestrogen formation and in phase II sulfation or glucuronidation in breast cancer etiology. *CYP1A1* and *CYP1A2* genotypes are associated with the generation of catecholestrogens, which have been associated with breast cancer risk [35]. In addition, *SULT1A1* and *UGT1A1* act on both estrogens and catecholestrogens, and therefore the potential effects of combinations of many genotypes of phase I catecholestrogen genes and phase II detoxification genes may identify mechanisms by which multiple genotypes in common pathways may influence breast cancer risk. In particular, the algorithm applied to empirical data supports the hypothesis that the formation of catecholestrogens in the context of the sulfation and/or glucuronidation of these compounds may be jointly associated with cancer risk. The combination of catecholestrogen metabolism genotypes and sulfation was previously identified in breast cancer risk [30] using standard logistic regression methods.

In AA endometrial cancer, only one high-scoring bi-clique was identified with a P-value of 0.0237. This may reflect the relatively small sample size in this group. This bi-clique involved the joint effect of CYP1A1*2C genotypes, CYP1B1*4 genotypes, and *SULT1E1* genotypes. *CYP1A1* and *CYP1B1* are involved in the generation of catecholestrogens, while *SULT1A1* is involved in the sulfation of both estrogens and catecholestrogens. In EA endometrial cancer, the highest scoring bi-clique was the main effect of CYP1A1*2C genotype. This effect was previously reported by our group[31] in analyses using traditional logistic regression methods. Therefore, the CHAMBER approach has identified the main effect of this genotype that was also identified by a standard analytical approach. The second highest scoring bi-clique observed here was the main effect of CYP3A4*1B genotypes, which are associated with increased catecholestrogen formation, and would therefore be expected to be associated with increased endometrial cancer risk. This association was also observed in our earlier paper [31]. This provides an additional assessment of the CHAMBER algorithm's ability to identify genotype combinations that may be involved in cancer risk that is an extension from our prior main effects or first order interaction explorations of our prior research [30].

### Etiologic Heterogeneity

When more than one pattern is found to have a strong effect on disease risk, it is relevant to ask whether the patterns represent variations of the same risk factor, or distinct risk factors (i.e., etiologic heterogeneity). Simulated dataset D4 was designed to model etiologic heterogeneity. Two disjoint genotype combinations were simulated (i.e., D2 and D3, Figure 2 and Table 2), such that enhanced risk could come from the D2 or D3 patterns. The effect of each risk-conferring pattern was simulated such that no additional risk was assigned to individuals who had both risk patterns. For D4 (Figure 2), one of the bi-cliques of interest (G03, G05) ranked first, but the second bi-clique (G01, G02, G04, G06), with comparable odds ratio and P-value, was ranked tenth.

**Figure 4:**
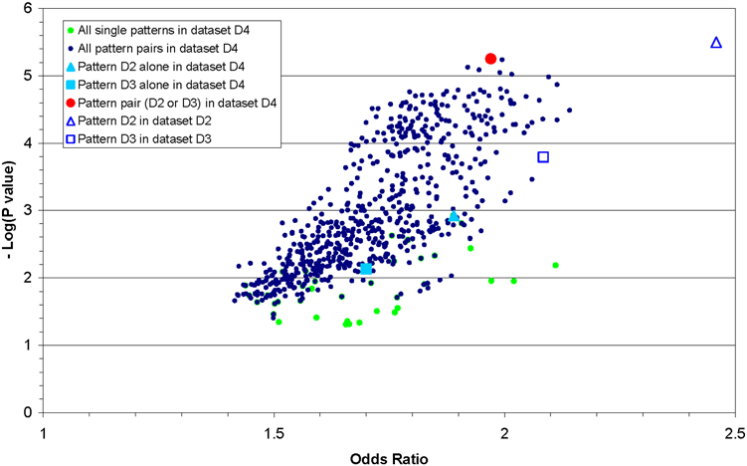
The designated pattern pair in dataset D4 is the highest scoring of all pairs. One of the components of the designated pattern (filled blue) could not be identified among the individual patterns in dataset D4 (green dots). The same two components (unfilled blue) scored much higher in single risk datasets D2 and D3.

Etiologic heterogeneity in a ranked list of patterns was also evaluated. Figure 4 shows the ORs and P-values for all pairwise combinations of the patterns with OR>1 and P<0.05, as found in D4 (black dots). Notie that the odds ratios and P-values for both designated patterns *alone* in dataset D4 (filled blue) are worse than the scores for the same patterns *alone* in the single-risk D2 and D3, respectively (hollow blue). This is because the 2×2 table for one pattern is skewed by the risk assigned to the other pattern, and *vice versa*. In D3, the people who do *not* have the pattern found in D3 but do have the pattern found in D2 had a 90% chance of being controls. When the pattern found in D2 also confers risk as in D4, those same people have only an 80% probability of being controls. Thus, counts are shifted from the on-diagonal quadrant to the off-diagonal quadrant of the 2×2 table, thereby reducing the OR.

In Figure 4, the score for the designated pattern pair “D2 or D3” in dataset D4 (red) is much higher than its individual components (filled blue). In fact, “D2 or D3” has the best score of all individual (green) and paired (black) patterns in dataset D4. The pair of patterns “D2 or D3” would not have been identified as the designated pattern by considering D2 and D3 alone because D3 ranked low for reasons discussed above. We were able to identify as “interesting” the signal from D3 by examining pattern pairs for high scores compared with the individual components.

**Figure 5:**
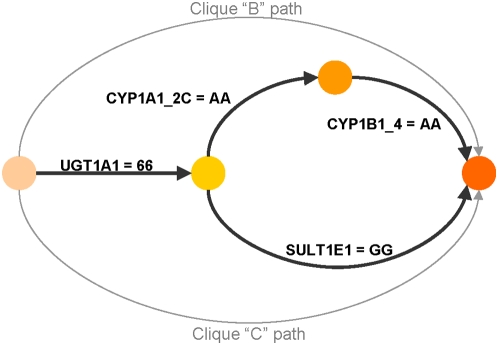
Motif suggested by pattern pair “BC” for a 3-gene pattern (“B”) and a 2-gene pattern (“C”) sharing 1 gene in a serial/parallel motif.

We also evaluated the potential for etiologic heterogeneity in the empirical dataset. We observed a combination in the AA breast cancer results that suggested the presence of etiologic heterogeneity. The three highest ranking patterns were A: (*UGT1A1* = *1*28 and CYP1B1*4 = AG), B: (*UGT1A1* = *1*1 and CYP1B1*4 = AA and CYP1A1*2C = AA) and C: (*UGT1A1* = *1*1 and *SULT1E1* = GG). We examined these 3 patterns for pairwise FOM and support overlap. Pairs AB and AC scored noticeably higher than their components, and had no support overlap, suggesting separate etiologies. Pair BC scored slightly lower than its components, and had 67% support overlap, suggesting that B and C are parts of the same etiology. Bi-clique B has three genes, bi-clique C has two genes, and they share one gene. Thus, the BC pair is consistent with a four-gene motif having two paths connecting common endpoints (a two-gene path in parallel with a three-gene path) with one shared segment (Figure 5).

## Discussion

The CHAMBER algorithm described here is an exploratory technique that searches the complete space of all combinations of putative risk factors and identifies the subset of features that are most likely to be of etiological interest. In both our simulated and empirical datasets, we show that the discovery of high-dimensional combinations and heterogeneity in the etiology of a complex disease may require not only computationally sophisticated algorithms but also careful examination and interpretation of the results of those algorithms to understand disease etiology. While in theory the CHAMBER algorithm can be applied with an unlimited number of genes and other risk factors, limited only by computational constraints, the main utility of this model may be to explore the effects of multiple genotypes and detect etiologic heterogeneity in complex disease. In addition, while the applications provided here focus on genetic variation, the application of both genes and environmental exposures can be explored using this algorithm. Given the numerous successes of genome-wide association studies, CHAMBER may be a useful tool for following up on the large number of “hits” from these scans. Here, we illustrate this potential by applying the model in a situation where main effects of genes had been previously identified, and use the CHAMBER algorithm to detect novel higher-order effects.

A key principle of association studies involving complex diseases, and a feature accounted for by CHAMBER, is that there is no one “correct” solution but instead there may be a set of solutions that describe the relationship between risk factors (e.g., genotypes) and disease. In our simulation results, the “correct” solution was in fact identified based on the simulated data. However, in real-life situations, it is possible that the algorithm may identify more than one highly ranked pattern, particularly when the signal from a bi-clique is weak. The results are nonetheless valuable in guiding subsequent validation studies and correlative laboratory experiments. Central to the application of this algorithm is to recognize when two bi-cliques actually represent the “same” allelic/genotypic combination. One metric used here is the percentage of people two bi-cliques have in common. For example, assume a bi-clique containing alleles A and B is associated with increased risk in a sample of 500 cases. If the number of cases with this pattern is 80 (i.e., 16% of all cases), the “signal” in this bi-clique may not be very strong. Nevertheless, pattern AB may rank very high using this algorithm, but its ranking may not be substantially different than the ranking of a bi-clique that contains A only. Assuming A and B are in linkage equilibrium, this would not be surprising if allele B were also frequent in the sample. Thus, we expect that the majority of the people who have pattern A also have pattern AB. These observations imply that when higher-order combinations exist in data, the algorithm may identify bi-cliques that include subsets of the alleles found in the higher-order combinations. Thus, to fully interpret the results of the CHAMBER algorithm requires not only the identification of high-ranking bi-cliques, but also an understanding of the lattice of relationships among bi-cliques.

We have compared the CHAMBER method with our previously published results analyzed by logistic regression [30] and by analyzing our simulated data by classification and regression trees (CART). In our earlier analysis of pairwise interaction using traditional logistic analysis methods, three significant first order interactions were observed. In European Americans, interactions between phase I *CYP1A1* genotypes and phase 2 sulfotransferase genotypes were observed in both AA and EA groups. Similar interactions were also observed in the present analyses using the CHAMBER approach, although additional potential higher order effects were detect by CHAMBER that were not seen in our earlier work (no prior multigene analyses of endometrial cancer have been undertaken).

The analysis of our simulated data (cases D2 and D4) using recursive partitioning as implemented as CART was not able to identify simulated patterns of higher order genetic associations when the lower order effects were weak. In particular, it only identified one of the 2 alleles seeded into D2 and did not identify any of the alleles in the 4-allele pattern seeded into D4. This result is not unexpected because CART is a greedy algorithm, which, as usually implemented, picks alleles one at a time that may affect the phenotype under study. In cases where the main effects derive from combinations of alleles and individual alleles do not give a strong signal, CART may miss important higher order effects. This was observed in the comparison of CHAMBER and CART provided here: CART may not be able to identify groups of alleles that are not individually identifiable. Even when they are, these effects could be masked by other alleles sending a stronger signal. However, CART (and other methods) may be an appropriate choice to identify combinations of alleles that are identifiable individually and in cases where they have a larger effect together.

Based on the results presented here, we believe the CHAMBER algorithm has value beyond other methods that have been proposed in this field. Our algorithm belongs to a broad class of algorithms called branch-and-bound methods, where an exhaustive decision tree of potentially exponential size (all combinations of variable values) is explored. CART has this property in that the nodes of the trees utilized in CART are explored in order of an objective function, with the best (strongest) effects being identified first. The tree is built and pruned by applying constraints which may include feasibility and objective function value. In the case of CART, one constraint is that the search proceeds greedily, at each step picking the best next step. This dramatically speeds up the search but also fails to explore much of the solution space. There are many situations in which this approach may succeed. However, CART approaches may miss higher-order relationships when there are no lower order effects (e.g., if the lower-order effects do not split out early in the construction of the tree). Unlike CART, CHAMBER is not a greedy algorithm (although it is possible to include greedy algorithm features), so it will find every combination. For problems of modest size, it explores the entire space of solutions. For larger problems, it carries out a directed search which, while pruned to respect realistic limitations on memory and running time, still carries out a search that is far more thorough than a greedy algorithm. While CHAMBER does not always carry out an exhaustive search of the solution space, which is potentially exponential in size, it does carry out a thorough search of the space in most realistic instances. In particular, it examines adding features to candidates in all possible orders, not just accepting one feature at a time as a greedy algorithm would do. When forced to prune the search space due to limitations on memory and time, it uses a directed search which favors good solutions over distinctly poorer ones. If the objective function is well behaved, in the sense that good solutions tend to contain good partial solutions, it will explore all attractive regions of the solution space and while it may miss the global optimum owing to the increasing coarseness of the search, it will not miss an entire good region. This together with the clustering (set covering) that is done following the original search, has proven sufficient to reveal the good candidate solutions in the problems we have studied. Compared with methods such as agglomerative clustering, our approach may be computationally more intensive, but it has the ability to search the solution space much more thoroughly and also allows us to recognize multiple clusters containing the same allele. This aspect of the CHAMBER approach corresponds to the realistic situation where an allele may be part of multiple pathways.

Furthermore, CHAMBER can find patterns that have a weak effect or are rare. However, combinations of risk factors (e.g., alleles) that are both rare and have weak effects may be missed. This is likely to be a limitation of most analytical methods. Finally, we do not compare our results against a common reference group. The FOM considered here is the comparison of a specific genotype combination vs. not having that combination. Since we don't compare to a single reference group, we cannot necessarily compare FOMs directly. FOMs can be compared across a single run in order to implement the set-covering algorithm, but it is not appropriate to compare FOMs across runs. Instead, we use the FOM as a measure of the strongest (e.g., most interesting) effect among all possible bi-cliques.

CHAMBER can be easily modified to meet individual research needs. First, CHAMBER was able to search the entire space of bi-cliques in our examples because the number of genotypes was limited. When the number of genotypes is large (e.g., in a genome-wide association study), CHAMBER can search the combinatorial space effectively, but may not be able to do so exhaustively. In those cases, computational constraints may be a limiting factor, and it may be important to consider several different FOMs in order to gain confidence that the search was sufficiently thorough. The growth of the number of candidate bi-cliques can be limited by carrying out a directed search[36], which can concentrate on expanding the most promising candidates. This approach is very flexible as we can set up a priority queue based on a figure of merit that combines the quality of the candidate (e.g., P value and odds ratio) and its position in the solution space. Note that, unlike classical search algorithms such as CART, this approach does not rely on the preceding nodes in a tree and is capable of searching the solution space uniformly or in a highly directed fashion. Second, we use the number of exposed cases (“support”) to filter our analyses. Other reference groups can be used without loss of generality. For example, unexposed controls are often used in epidemiological studies, and filtering to remove potentially underpowered bi-cliques can be done. This “in-line” filtering is used to reduce the computational load and output complexity by avoiding exploring branches of the solution space that are deemed *a priori* to be uninteresting. For example, the analyst may choose the limits that can be put on the bi-clique support to meet the needs of a particular anlaysis. Third, CHAMBER allows the user to adjust the stringency of the analysis by altering (or removing) the filter and parameter values. Similarly, while we consider CHAMBER to be an exploratory algorithm, the use of P-values may require correction for multiple testing by a variety of standard means [37]. Finally, the algorithm was explored by using a pathway of candidate genes. However, CHAMBER can also be used in studies of multiple exposures and/or genes.

## Methods

### Algorithm

Our approach assumes here a case-control sample ascertained using appropriate epidemiological study design methods. The basis for this approach are discrete math principles of graph theory [38], and have been previously described by Mushlin et al. [36]. We define a *node* to be a person or a value of a *characteristic*, which is a risk factor (e.g., genotype). A *bin* is a set of values (e.g., an allowed value or collection of values) of a characteristic. We refer to the values of the data associated with each person as *features*. An *edge* is a connector between a person node and a bin node containing one or more features. *Adjacency* refers to two nodes connected by an edge. There are two ways to represent the relationships of interest: An *adjacency matrix* (also known as an *edge table*) is a matrix of relationships between nodes. A *graph* is a pictorial representation of the relationship among nodes. Using these definitions, a *clique* is a sub-graph in which all nodes are connected. A *bi-clique* is a sub-graph where all nodes of one kind (e.g., people) are connected to all nodes of another kind (e.g., genotypes). In a bi-clique, all people nodes are connected to all bin nodes, but people nodes are not connected to other people nodes, and bin nodes are not connected to other bin nodes.

The goal of CHAMBER is to reveal all possible bi-cliques of interest and to prioritize bi-cliques that are of greatest interest. In many cases, such as those presented in this paper, we do. (We detect and report pruning as it occurs.) It is inevitable that we will generate some false positives. It is important, however, to note the difference between bi-cliques that are totally false and bi-cliques which are either overly specific (too many features) or overly general (too few features). By clustering solutions (set covering), we select the most parsimonious feature set to represent a collection of nested bi-cliques. False Discovery Rate analysis can be used to estimate false positives of the traditional sort. In the end, domain knowledge can be used to prune some of the false positives. Finally, we do not claim that Chamber is sufficient alone to completely solve the problem. In the end, the most promising candidates must be evaluated in the laboratory. We do claim that Chamber can significantly reduce the number of candidates to so examine, without eliminating useful ones to explore.

The five steps outlined below, in Figure 1, and Appendix S1 are analogous to those of tree-building and pruning seen in recursive partitioning algorithms[2], but are not limited by the order in which branches are added or removed from the tree. Figure 1 provides an overview of the algorithm.

We chose to select for further analysis those bi-cliques with “good” P-values that implied risk and that had a “well-behaved” 2×2 Table. This was achieved by selecting bi-cliques with P-value<0.05, odds ratio>1, and N_min_ = 2, where N_min_ is the smallest cell in the 2×2 Table. For each selected group of discovered bi-cliques, a cost matrix is constructed as input to the set covering algorithm[33] (Appendix S2) The output is a list of explanatory feature sets used and a list of the bi-cliques they explain. This list of explanatory feature sets is taken to be the most parsimonious description of the many overlapping patterns detected in the original dataset.

Finally, etiologic heterogeneity is inferred based on the disjointedness of the identified patterns (Appendix S3). In particular, patterns with distinct groups of people and distinct groups of features suggest distinct etiologies. Two (or more) distinct groups of features may also be present in a single group of people who have a significantly higher risk than people having either of the groups of features alone. A measure of the overlap in support (or features) between two bi-cliques is the Jaccard index[39], *H* = *[C1∩C2]/[C1∪C2]*, where *C1* is the support (or feature) set of bi-clique 1 and *C2* is the support (or feature) set of bi-clique 2. Small values of *H* suggest distinct etiologies.

### Simulated Data Example

Simulated data were generated using a macro program coded in SAS v 9.0 in order to evaluate the behavior of the CHAMBER algorithm in candidate gene association studies involving high-dimensional data. Simulated data were generated for eight genes assumed to be in Hardy-Weinberg equilibrium. These simulated data were intended to reflect frequently encountered empirical allele and genotype frequencies, including unknown (missing) genotypes. Specifically, we generated these simulated data to reflect the data observed in the WISE study [30], [31], [32]. For each dataset, 500,000 individuals were generated to model a variety of genetic risk scenarios involving one or more genotypes conferring an enhanced risk of disease. We specified disease risk for all possible multi-locus genotypes in the simulated cohorts. A baseline disease risk of 10% was assigned to all genotype categories. The multi-locus combined risk for each of the combinations of genotypes at up to 8 loci was updated to specify the relative risk associated with a given joint genotype. Case/control designation was assigned by comparison of the combined computed disease risk to a random number generated from the standard uniform distribution. Case status is determined by determining probability of disease for those with a particular genotype combination to be 20% or greater. A random number is then assigned to each individual, and if that number is less than 0.2, then we assign is “case” status; those with combined risks greater than the random number were assigned control status. Random sampling with replacement of the simulated cohort was performed to create subsets of case/control groups which were used as input to the bi-clique finding algorithm.

### Empirical Data example: The WISE Study

To further evaluate the ability of the bi-clique-finding model to identify combinations among genotypes as they may influence disease risk, we employed data from the WISE study, a population-based study of breast and endometrial cancer risk [31], [40]. From the total WISE sample set, we have studied 225 African American and 613 White breast cancer cases, who were compared to 512 African American controls and 820 White controls. In addition, we studied 44 African American and 462 White endometrial cancer cases compared with 329 African American and 1,082 White controls who participated in the WISE study. Using genes involved in the downstream metabolism of estrogen, we chose one SNP in each of eight genes that are thought to have a functional effect on hormone metabolism and/or cancer risk in order to illustrate the CHAMBER algorithm. The variants studied were: *COMT* Val158Met (rs4680), *CYP1A1* Ile462Val (*2C; rs1048943), CYP1A2*1F (rs762551), *CYP1B1*, (Asn452Ser, *4; rs1800440), CYP3A4*1B (rs2740574), *SULT1A1* Arg213His (*2; rs9282861), *SULT1E1* -64G>A Promoter Variant (rs3736599), and variants in UGT1A1 (*28). These variants were assayed as previously described [31].

### CART Analysis

We implemented CART analysis using the Java version of Quinlan's C4.5 algorithm [41] called J48, as implemented in the WEKA software [34]

### Software

The software is written in Java and should run with little or no modification on most OS's. The data format is simple flat files (e.g., .csv files) with defined row and column semantics. There is a command line interface to all the programs, and the overall process involves running a small number of programs in sequence. IThe method will easily scale to dozens of SNP's, a useful range for candidate gene studies, follow up of GWAS results, or other similar studies in which genotypes have strong main effects or when main effects are weak or non-existent but important higher order effects exist. The computational complexity is near-linear in the number of candidate partial solutions explored, but that number can grow exponentially with the number of SNPs. In-line filtering, such as support or feature count thresholds, can be used to extend the practical range. If the landscape of solutions can be estimated, the FOM measure can be selected to optimize the candidates kept in the queue with respect to that landscape. Beyond that, it is necessary to decompose the problem into (possibly overlapping) sets of SNPs and to then make multiple runs. By iterating through promising solutions, it is possible to explore somewhat larger problem spaces. Parallel processing could be used to advantage in exploring such decomposed problems. CHAMBER as currently implemented is not intended for genome wide studies.

## Supporting Information

Appendix S1 (0.11 MB DOC)

Appendix S2 (0.11 MB DOC)

Appendix S3 (0.11 MB DOC)
