# Supplementary material for: Clique-Finding for Heterogeneity and Multidimensionality in Biomarker Epidemiology Research: The CHAMBER Algorithm
Source: PLoS One. 2009 Mar 16;4(3):e4862. doi: 10.1371/journal.pone.0004862 (PMC2653643; doi:10.1371/journal.pone.0004862)
Supplement: Appendix S1 — (0.11 MB DOC) [file pone.0004862.s001.doc]

# Appendix S1: Detailed Clique-Finding Algorithm Description

*Step 1: Bipartite Graph Construction*

The algorithm first involves bipartite graph construction to identify all relationships between nodes (Figure 1, Phase I). A bipartite graph is constructed by creating an edge between the subject (s) and a genotype (t). We construct a bipartite graph, G = (S,T,E), where S and T are disjoint sets of nodes and E is a set of undirected edges, e = (s,t), where s (i.e., a source) is in S and t (i.e., a terminal) is in T. Our goal is to find all maximal bi-clique sub-graphs B = (S_B_, T_B_ , E_B_ ) of G, where S_B_, T_B_, and E_B_ are subsets of S, T, and E, respectively, and there is an edge e = (s_B_ , t_B_ ) for all pairs of nodes in S_B_ and T_B_. A bi-clique, B, is said to be maximal if there is no other bi-clique B’= (S’_B_ ,T’_B_ , E’_B_), where S_B_ is a subset of S’_B_, or T_B_ is a subset of T’_B_.

*Step 2: Maximal Bi-clique Formation*

In the second step, the algorithm undertakes maximal bi-clique formation by exhaustively searching the entire space of all genotype combinations to identify an initial set of maximal bi-cliques (Figure 1, Phase II). We start with two sets of candidates which we refer to as s-cliques and t-cliques. For each s in S, we form an s-clique, C(s) = [[s], T(s)] where T(s) is the set of t such that there is an edge (s,t) in E. Similarly, for each t in T we have a t-clique C(t) = [S(t), [t]]. All the candidate bi-cliques we identify can be described as generalizations of this form. Specifically, given any set A of sources, we have an s-clique C(A) = [A , T(A)] where T(A) is the set of t, such that there exist edges (s,t) for all s in A. Similarly, we have t-cliques C(B) = [S(B) , B]. The basic operation which is used to expand s-cliques is to merge pairs of bi-cliques C(A_1_) and C(A_2_) such that C( A_1_ ∪ A_2_ ) = [S(A_1_ ∪ A_2_ ) , T(A_1_) ∩T(A_2_)]. Similarly, the basic operation to expand t-cliques is C( B_1_ ∪ B_2_ ) = [S(B_1_) ∩ S(B_2_) , T(B_1_ ∪ B_2_ )].

*Step 3: Figure of Merit*

In the third step, a Figure of merit (FOM) is generated to prioritize “interesting” bi-cliques (Figure 1, Phase II). The FOM can be any measure inherent to the data. Here, we consider values of features (e.g., genotypes) in a 2x2 contingency Table with affected cases and unaffected controls contingent on exposure (e.g., genotype). Using standard methods, we can then compute a variety of FOM values from the 2x2 Table, including the prevalence of the exposure in a particular bi-clique, the number of features in a bi-clique, the odds ratio (OR), the relative risk (RR), the attributable risk (AR), and others. The FOM can be further expanded in definition to consider more complex metrics, including OR, RR, or AR values weighted by the frequency of observations in a node (e.g., the “support” defined as the frequency of exposed cases), or by the P-value. Note that P-value, when used as a FOM, is a relative measure used to rank the bi-cliques, rather than an absolute measure to reject a hypothesis. The P-value is a convenient measure of the asymmetry of the 2x2 Table. The OR gives a very similar rank order as the P-value. We did use an absolute P-value < 0.05 and absolute OR > 1.0 to retain the most interesting candidates for further analysis, but the set covering process (see Step 5) is minimally affected by these low ranked candidates. While not computed here, statistical significances associated with FOMs can be obtained empirically by random permutation of the case status of individuals to obtain the empirical distribution of FOM values.

*Step 4: Lattice Building*

In the fourth step, a “lattice building” step uses the set of expanded maximal bi-cliques, obtained as described above (Figure 1, Phase III). This lattice is formed by connecting each pair of bi-cliques to their least upper bound and their greatest lower bound using principles of set union and intersection. The bi-clique problem is NP-complete, and the actual number of maximal bi-cliques in a graph may be exponentially large. If a bi-clique has n sources and m terminals, then there are 2^n^2^m^ smaller bi-cliques from which it could be built. Because the total number of potential bi-cliques is large, it is possible to impose criteria to limit the number and scope of bi-cliques to be considered. First, bi-cliques can be considered only if they contain a minimum number of people. Second, the number of bi-cliques can be limited by starting with large C_S_ and C_T_ and then decreasing those values, or by decreasing C_S_ while increasing C_T_ (and vice versa). Third, as we expand an s-clique, we intersect its terminal set with other terminal sets, so the resulting terminal set never gets larger. Therefore, if T(A) has fewer than C_T_ elements, then we can eliminate A from further consideration as a source set in an s-clique. A similar rule is applied to t-cliques. Fourth, cliques can be removed that are known to not be maximal. Thus, if we already have a maximal candidate C(S_1_,T_1_) and we generate a candidate C(S_2_,T_2_), where S_2_ is contained in (or equal to) S_1_, and T_2_  is contained in (or equal to) T_1_, then C(S_2_,T_2_) is dominated by C(S_1_,T_1_) and can be removed from further consideration. The algorithm checks these conditions every time it generates a new candidate bi-clique and limits the list of candidate bi-cliques. While not applied here, lexicographic ordering could be imposed to avoid generating the same candidate node in more than one way. For example, the source set [ s_1_ , s_2_ , s_3_ ] could be generated by adding s_3_ to [ s_1_ , s_2_ ] or by adding s_1_ to [s_2_ , s_3_ ], etc. In addition, only source bi-cliques that can lead to a source that is associated with at least one terminal in the terminal set of the candidate are used to expand the candidate. That is, given an s-clique C(A,B), its feasible neighbor set contains all s such that s is in S(t) for at least one t in B. Analogous neighbor sets are generated for the t-cliques. Fifth, s-cliques (t-cliques) can be prioritized using various measures such as the absolute size of the source (terminal) set, the size of the source (terminal) set relative to the constraint on it, the lexicographic value of the source (terminal) sets, or other metrics associated with the bi-clique. In addition, candidates can be limited to one type of bi-clique (i.e., s-cliques or t-cliques) and used as the sole basis for bi-clique expansion. Finally, for all sibling nodes, a new node can be created that represents the union of all sibling nodes.

The result of these first four steps is the subset of all acceptable bi-cliques that form a filtered lattice, which is a collection of subgraphs of the full lattice. The nodes of the lattice are the bi-cliques (Figure 1, Phase IV). An edge connects two bi-cliques, parent A and child B, if and only if S_A_ is a subset of S_B_ and T_B_ is a subset of T_A_. We define the *lineage* of a bi-clique as a unidirectional child-to-parent path from the given bi-clique up to a bi-clique with no parent. By construction, traversing a lineage in one direction deletes features from the source set and adds people to the terminal set, and *vice versa* for the opposite traversal, independent of the data from which the lattice was derived.

*Step 5: Optimal Bi-clique Identification*

In the fifth step, the bi-cliques of greatest interest are identified using a parsimony principle by which “optimal” bi-cliques should contain the most parsimonious set of features, and the addition of more features does not substantially improve the FOM. To achieve this, we employ the set covering approach[1] (Appendix S2). This approach requires the identification of a set of explanatory features [v_j_] and bi-cliques [q_j_] determined as described above. Bi-cliques are first filtered by using the output of the bi-clique discovery algorithm[2]. Filter parameters include P-value, odds ratio (OR), and minimum count in any of the 4 cells of the 2x2 Table (N_min_). The purpose of the N_min_ constraint is to filter out bi-cliques that have artificially large OR due to very small numbers. We typically use filter parameters of P-value < 0.05, OR > 1, and N_min_ = 2. This filtered set of bi-cliques is assigned to both the [v_j_], and to the [q_j_]*.* In order to identify the optimal solution, and to avoid placing constraints on the solution that may result in missing important relationships, we use [q_j_] = [v_j_]. We then assign to each bi-clique k, where k ∈ [q_i_] or k ∈ [v_j_], a *score* S(k) = FOM(k) = −log_10_(P(k)). This is the same FOM that is used to prioritize the queue in the bi-clique discovery algorithm (see above). Other scoring systems are possible, but -log_10_(P) measures only statistical significance without regard to explicit risk measures.

In order to decide which sets of explanatory features v_j_ are reasonable to consider, we propose a “cost” for the use of each v_j_. We proceed to scale all costs to be dimensionless quantities on the interval [0,1]. Thus, we assign values on the interval [0,1] for each of the following costs:

c_ij_ = cost of explaining bi-clique q_i_ with explanatory feature v_j_.

c_j_ = one-time cost of using a particular v_j_ in the solution;

c_im_ = cost of *not* covering bi-clique q_i_ with *any* element of [v_j_];

c_m_ = one-time cost of *not* covering at least one bi-clique.

As a guide in constructing a cost model, note that the overall purpose of the set covering operation is to strike a balance between high scoring bi-cliques with narrow support (e.g., those feature sets with high FOM scores but that are rare and therefore are associated with a low attributable risk), and more widely supported bi-cliques having lower scores (e.g., those feature sets with low to moderate FOM scores but that are common and therefore are associated with a high attributable risk). To achieve such a balance, the cost model should have the following general properties:

1. The cost of explaining a high scoring bi-clique q_i_ (e.g., one with a high FOM) with a low scoring v_j_ increases with the difference in the FOM scores.
2. Explaining a bi-clique having a lower FOM than that of the v_j_ can be done at no cost.
3. The cost of *not* choosing to explain a bi-clique with *any* set of explanatory variables v_j_ increases with the FOM of the bi-clique.

In keeping with these principles, the costs are modeled to explain bi-cliques q_i_ with v_j_ (c_ij_), and to intentionally not consider q_i_ (c_im_), using the following rules:

1. c_ij_ = (S(q_i_) - S(v_j_)) / S(q_i_) for S(q_i_) ≥ S(v_j_);
2. c_ij_ = 0 for S(q_i_) < S(v_j_);
3. c_im_ = S(q_i_) / max [S(q_i_)].

In addition, the following rules are imposed for those c_ij_ that are not allowed because v_j_ is not a subset of q_i_, and for fixed costs of both using v_j_ (c_j_) and using v_m_ (c_m_), defined as a set of explanatory features not contained in c_j_:

1. c_ij_ = 1 for v_j_ not a subset of explanatory features contained in q_i_;
2. c_j_ = 1 for all v_j_ except v_m_;
3. c_m_ = 0 for v_m_.

The value of c_m_ relative to c_j_ determines the fraction of the bi-cliques that can be explained by v_j_. When c_m_ / c_j_ >> 1, all bi-cliques are explained by one or more sets of explanatory features, since the cost of not explaining even one bi-clique is very high. As c_m_ / c_j_ decreases, it gets “cheaper” to not explain some bi-cliques, namely those with low c_im_. When c_m_ / c_j_ = 0, no bi-cliques are forced to be explained. In this situation, bi-cliques are explained (or not) based only on c_ij_ (since all c_j_ are the same). We let c_m_ = 0 to avoid including explanatory feature sets in the solution that only explain low scoring bi-cliques. The cost to cover a bi-clique having an equal or lower FOM is zero. Thus, the cost to cover a bi-clique having a higher FOM is proportional to the difference. The algorithm does not require that every bi-clique be covered, but there is a cost associated with skipping a bi-clique. Our model assigns a cost-to-skip proportional to the FOM of the bi-clique to be skipped.

For each filtered set of discovered bi-cliques (e.g., P-value < 0.05, OR > 1, N_min_ = 2), a cost matrix is constructed as input to the set covering algorithm[1]. The output is a list of explanatory feature sets used and a list of the bi-cliques they explain. This list of explanatory feature sets is taken to be the most parsimonious description of the many overlapping patterns detected in the original dataset.
