# Supplementary material for: Clique-Finding for Heterogeneity and Multidimensionality in Biomarker Epidemiology Research: The CHAMBER Algorithm
Source: PLoS One. 2009 Mar 16;4(3):e4862. doi: 10.1371/journal.pone.0004862 (PMC2653643; doi:10.1371/journal.pone.0004862)
Supplement: Appendix S2 — (0.11 MB DOC) [file pone.0004862.s002.doc]

# Appendix S2: Set Covering Algorithm

The goal of the Set Covering Algorithm[1] is to identify the most parsimonious configuration of bi-cliques that provide optimal explanation for the multifactorial relationship between genotypes and disease. In the Set Covering Problem (SCP), we are given a set of objects, in this case bi-cliques, q_i_. We are also given a set of explanatory feature sets, v_j_. In this case, the v_j_ are also bi-cliques but with a smaller or equal number of features. There is a cost, c_j_, associated with each of the v_j_ and a cost c_ij_, associated with explaining q_i_ with v_j_.

Our objective is to explain all the q_i_ at minimum cost. Let the variable x_ij_ be 1 if we explain q_i_ with v_j_ and 0 otherwise. Similarly, let the variable y_j_ be 1 if v_j_ is part of our solution and 0 otherwise. Thus, formally, we have the problem:

P: Minimize z = Σ _ij_ c_ij_ x_ij_ + Σ _ij_ c_j_ y_j_ (1)

subject to: Σ _j_ x_ij_ = 1 for i [ i.e., cover all q_i_]

x_ij_ ∈ y_j_  i, j [ i.e., include v_j_ if it covers q_i_]

x_ij_ ∈ [ 0 , 1 ] i, j

y_j_  ∈ [ 0 , 1 ] i

This is an Integer Programming problem (IPP) that is a combinatorial optimization problem in which a subset of a discrete set of objects must be selected. To solve this problem, we use a “relaxation” heuristic [1] to solve the problem. Given an IPP, we can further constrain this problem with a constraint set and an objective function (i.e., the function we wish to minimize) and create a new problem, IPP_R_, a relaxation of IPP, by loosening (relaxing) some of the constraints, possibly by replacing the objective function (i.e, z in equation 1) by another function that represents a lower bound on the original objective function. Thus, any solution to IPP is still a solution to IPP_R_ (although the converse is not necessarily so) and the value of the optimal solution to IPP_R_ is a lower bound on the value of the optimal solution to IPP. Thus, we replace z by a function that yields a lower bound.

The relaxation proposed here is based on the observation that if the y_j_‘s were all zeros, we would simply find, for each i, the minimum over j of c_ij_ and then set x_ij_ to 1 for that j. This bound can be tightened significantly by observing that the cost of the covers can be distributed among the bi-cliques being covered by them. In particular, we define variables p_ij_, to be penalties to be added to the c_ij_ and recast the SCP as IPP_R_: Minimize z = Σ _ij_ [ (c_ij_+p_ij_) x_ij_ ], subject to the following constraints: Σ _j_ x_ij_ = 1 for i (i.e., cover all q_i_), Σ _i_ p_ij_ < c_j_  i (i.e., distribute the c_j_ over the q_i_), x_ij_ > 0, y_j_ > 0, and p_ij_ > 0. In this case, the optimum solution is to find for each i the smallest c_ij_ + p_ij_ and set x_ij_ to 1 for that j. Note that this gives a lower bound regardless of how we assign the p_ij_, since the sum of the p_ij_ can be no larger than the c_j_’s we have removed. In particular, consider the following solution values:

z*[IPP] = the optimal solution to the original problem IPP (2)

z*[IPP_R_] = the optimal solution to IPP_R_ (3)

z+[IPP_R_] = the solution to IPP_R_ using the same covers as in solution z*[IPP].

Thus,

z*[IPP_R_] < z+[ IPP_R_] < z*[IPP]

Equation (2) comes from the fact that z*[IPP_R_] is the optimal solution to IPP_R_ and hence has a value no larger than any other solution to IPP_R_. Equation (3) comes from the observation that a solution to IPP_R_ accounts for only part of the cost of the covers involved; i.e, the cost of covers, j, for which at least one x_ij_ is set to 1.

We are free to set the p_ij_ to have any value, subject to the constraints imposed on them. We would like to set them to values which make the lower bound, z*[IPP_R_], as tight as possible. To do this, we would like to make the minimum value of c_ij_+p_ij_ for each i as large as possible, since the optimal solution to IPP_R_ is the sum of the minimum values of the c_ij_+p_ij_ for each i. Note that, starting with all p_ij_ set to zero, setting a p_ij_ to a non-zero value does not increase the minimum of c_ij_+p_ij_ for the given i unless c_ij_ was the smallest value for that i and even then, the minimum will increase only until the next smallest value of c_ij_ is reached. Thus we solve IPP_R_ is as follows. For all i and j, set x_ij_ , y_j_ and p_ij_ to 0. For each i, let z_i_ be the cost associated with q_i_. This is in general equal to min_j_ [ c_ij_+p_ij_ ]. Initially, z_i_ = min_j_ [ c_ij_ ]. z, the current lower bound, is defined as z = Σ _i_ [ z_i_ ]. Let k be the number of p_ij_ we are willing to adjust at one time for any i in order to increase z_i_. As explained above, in an effort to keep the lower bound as tight as possible, we prefer to keep k as small as possible. Initially, k is set to 1. Let s_j_ be the slack associated with v_j_, defined to be the unassigned part of c_j_. In general, s_j_ = c_j_ - Σ [ p_ij_ ]. Initially, s_j_ = c_j_. In order to maintain the validity of the lower bound, we require that s_j_  0. If for any j, s_j_ reaches 0, we add v_j_ to the solution. Let z_old_ be the previous value of z. Set z_old_ = -1. We now proceed with the following algorithm:

Step 0: Initialize k, p_ij_, z_i_, z, z_old_ and s_j_ as described above.

Step 1: If z = z_old_, go to Step 3. Otherwise,

Set z_old_ = z

Set k = k+1

Step 2: For each i,

Step 2.1: Let δ_i_ Let be the maximum amount we are willing to increase z_i_.

Set δ_i_ = c_i_[k] - z_i_ , where c_i_[k] is the k^th^ smallest value of c_ij_

Step 2.2: Let δ_i_ be the maximum amount we are actually able to increase z_i_.

Set δ_i_ = min_j_ [ s_j_ + (c_ij_ + p_ij_ -z_i_ ) ]

This constraint comes from the facts that we cannot let any s_j_ become negative and in order to actually increase z_i_ , we will need to increase p_ij_ to the extent that c_ij_ + p_ij_ is less than z_i_ .

Step 2.3: Set z_i_ = z_i_ + min [δ_i_ , δ_i_ ]

Set z = z + min [δ_i_ , δ_i_ ]

For all j, set p_ij_ = min [ 0 , z_i_ - c_ij_ ]

Set s_j_ = c_j_ - δ_i_ [ p_ij_ ]

Return to Step 1

Step 3: For each j, if s_j_ = 0, set y_j_ = 1

For each i, set x_ij_ to 1 for some j such that y_j_ = 1 and c_ij_ is as small as possible.

The actual cost of the solution obtained in this way is z[P] = Σ _ij_ c_ij_ x_ij_ + Σ _ij_ c_j_ y_j_. The lower bound obtained is z[P_R_] = Σ _ij_ [ (c_ij_+p_ij_) x_ij_ ]. The difference (gap) is an upper bound on how far the solution is from the optimum. If the gap is non-zero, it is sometimes possible to improve the actual solution by dropping one or more of the covers and reassigning the bi-cliques which were assigned to it. We would, of course, assign these bi-cliques to the remaining selected cover, j, with minimum c_ij_. In practice, it is possible to use a simple, greedy algorithm to do this:

Step 1: For each selected cover, j, compute p_j_, the profit associated with dropping v_j_:

Let S_j_ = [ i | x_ij_ = 1 ] ; i.e., the covers currently assigned to v_j_

Let c_i2_ = c_ik_ where v_k_ is the next best (selected) cover to assign bi-clique q_i_ to.

p_j_ = c_j_ - Σ _i in Sj_ [c_i2_]

Step 2: If j p_j_ < 0 , stop. Otherwise drop v_j_ from the solution, resassign the covers assigned to it, and return to step 1. It is also possible to assign a cost, c_iN_, to not covering bi-clique q_i_ at all. To model this, we simple add a new “cover”, v_m_, with c_m_ = 0 and c_im_ = c_In_
