# Supplementary material for: Clique-Finding for Heterogeneity and Multidimensionality in Biomarker Epidemiology Research: The CHAMBER Algorithm
Source: PLoS One. 2009 Mar 16;4(3):e4862. doi: 10.1371/journal.pone.0004862 (PMC2653643; doi:10.1371/journal.pone.0004862)
Supplement: Appendix S3 — (0.11 MB DOC) [file pone.0004862.s003.doc]

**Appendix S3: Effect of etiologic heterogeneity on odds ratios**

Table A1 demonstrates the effect of etiologic heterogeneity on odds ratios using two disjoint patterns for clarity. Consider pattern P1. In a population where there is no other risk pattern, such as occurs in dataset D2, the odds ratio is given by the usual formula (example 1). The same is true for pattern P2, analogous to dataset D3 (example 2). But when either P1 or P2 (or both together) have risk, the odds ratio can be expressed in terms of the counts of the component patterns. If we look only at one of the components, say, P1, as if it were the only risk factor, we see that the counts in cell c increase, and those in cell d decrease, relative to the counts when P1 *was* the only risk factor. Some of the people who did not have P1, specifically those with P2, had a good chance of being controls in the P1-only risk environment. But when P2 is also a risk, a2 of those people shift from controls to cases. In the P1 table this occurs in the bottom row and yields the counts shown (example 3). The symmetric shift occurs when considering P2 alone (example 4). The effect is seen in the simulated data (Figure 4) as a drop in odds ratio from the unfilled blue to the corresponding filled blue points.

Example 5 shows the effect of a P1-or-P2 risk, meant to model etiologic heterogeneity. Assuming the case and control totals (column sums) are constant, and recognizing that the counts for the top row are the sums of the top row counts for P1 and P2, we can complete the table and compute the odds ratio as shown. Note that the value of this expression relative to the other four examples could be higher or lower, depending on the actual counts. In general we observed the odds ratio of most pairs to be slightly higher than the individual components.

# Table A1: Examples of odds ratios for individual patterns and pattern pairs. Odds ratios for individual patterns in a heterogenious etiology (examples 3 and 4) are always lower than the corresponding patterns in a single etiology (examples 1 and 2). When considered together in a heterogeneous etiology however, (example 5), the odds ratio for the pair can be higher or lower, depending on the how the risk is distributed in the population.

| ***Example*** | ***Patterns P1 and P2 are Disjoint*** |  | ***Cases*** | ***Controls*** | ***Odds Ratio*** |
| --- | --- | --- | --- | --- | --- |
|  |  |  |  |  |  |
| *1* | *Only P1 carries risk* | P1 | a1 | B1 | (a1*d1) / (b1*c1) |
|  | *as in dataset D2* | not P1 | c1 | D1 |  |
|  |  |  |  |  |  |
| *2* | *Only P2 carries risk* | P2 | a2 | B2 | (a2*d2) / (b2*c2) |
|  | *as in dataset D3* | not P2 | c2 | D2 |  |
|  |  |  |  |  |  |
| *3* | *P1 or P2 or both carry risk* | P1 | a1 | B1 | (a1*(d1-a2)) / (b1*(c1+a2)) |
|  | *as in dataset D4* | not P1 | c1+a2 | d1-a2 |  |
|  |  |  |  |  |  |
| *4* | *P1 or P2 or both carry risk* | P2 | a2 | B2 | (a2*(d2-a1)) / (b2*(c2+a1)) |
|  | *as in dataset D4* | not P2 | c2+a1 | d2-a1 |  |
|  |  |  |  |  |  |
| *5* | *P1 or P2 or both carry risk* | (P1-or-P2) | a1+a2 | b1+b2 | ((a1+a2)*(d1-b2)) / ((b1+b2)*(c1-a2)) |
|  | *as in dataset D4* | not (P1-or-P2) | c1-a2 | d1-b2 |  |

1. Erlenkotter D (1978) A Dual-Based Procedure for Uncapacitated Facility Location. Operations Research 26: 992-1009.

2. Mushlin R, Kershenbaum A, Gallagher S, Rebbeck T (2007) A graph-theoretical approach for pattern discovery in epidemiological research. . IBM Systems Journal In Press.
